# Inhibitory effects of pentoxifylline on inflammation-related tumorigenesis in rat colon

**DOI:** 10.18632/oncotarget.26119

**Published:** 2018-09-21

**Authors:** Yohei Shirakami, Takahiro Kochi, Masaya Kubota, Hiroyasu Sakai, Takashi Ibuka, Kazuto Yoshimi, Takashi Kuramoto, Takuji Tanaka, Masahito Shimizu, Mitsuru Seishima

**Affiliations:** ^1^ Department of Informative Clinical Medicine, Gifu University Graduate School of Medicine, Gifu 501–1194, Japan; ^2^ Department of Gastroenterology, Gifu University Graduate School of Medicine, Gifu 501–1194, Japan; ^3^ Genome Editing Research and Development Center, Graduate School of Medicine, Osaka University, Osaka 565–0871, Japan; ^4^ Institute of Laboratory Animals, Graduate School of Medicine, Kyoto University, Kyoto 606–8501, Japan; ^5^ Department of Pathological Diagnosis, Gifu Municipal Hospital, Gifu 500–8513, Japan

**Keywords:** colorectal cancer, pentoxifylline, inflammatory bowel disease (IBD), oxidative stress

## Abstract

Chronic inflammation in the colorectum increases the risk of colorectal cancer development. Pentoxifylline, a medicine used for improving the circulation, has been reported to inhibit TNF-α production and to ameliorate inflammatory bowel disease and non-alcoholic steatohepatitis. In this study, we investigated the effects of pentoxifylline on inflammation-related colon tumorigenesis in a rodent model using Kyoto APC delta rats, which have APC mutation and are susceptible to colon carcinogenesis. Male Kyoto APC delta rats were treated with azoxymethane and dextran sodium sulfate, and were subsequently administered water, with or without pentoxifylline. At the end of the experiment, the development of colorectal tumor was significantly inhibited in the pentoxifylline group. The pentoxifylline treatment also lowered the levels of oxidative stress markers and mRNAs of pro-inflammatory cytokines, including TNF-α and IL-6, in the colon mucosa. The PCNA labeling index and the inflammation score were also decreased in the colon of rats in the pentoxifylline -treated group. We also used an endoscopy to observe the tumor progression and inflammation in the colon of rats, revealing that inflammation grade was significantly lower in pentoxifylline-treated group at several points during the experiment. These findings suggest that pentoxifylline treatment might be useful for chemoprevention of inflammation-related colon cancer.

## INTRODUCTION

Colorectal cancer (CRC) is a common malignant disease with high mortality. The clinical incidence of CRC has increased gradually over the past decade, and it is considered as one of the most important health issues worldwide [1]. This warrants increased attention on prevention strategies, in addition to early detection and treatment of high-risk CRC patients.

CRC is a heterogeneous disease attributed to multiple genetic mutations and epigenetic alterations involving genes regulating cellular growth and differentiation [2]. Chronic inflammation is one of the key predisposing factors in the development of CRC, and is a major complication in inflammatory bowel diseases (IBD), including ulcerative colitis and Crohn's disease [3, 4]. Patients with IBD are considered to be at high risk for CRC and need careful follow-up [5]. The primary prevention of CRC in IBD patients has attracted a lot of attention, since the clinical characteristics of IBD suggest that patients with the disease are appropriate targets for chemoprevention of CRC. In several clinical studies, 5-aminosalicylic acid (ASA), an anti-inflammatory drug, has been shown to protect against CRC development in patients with IBD [6, 7]. This indicates that the agents targeting inflammation-associated molecules can suppress the development of IBD-related CRC.

Pentoxifylline (PTX) is a methylxanthine derivative, which works as a competitive non-selective phosphodiesterase inhibitor. It is used as a medicinal agent for amelioration of circulation in peripheral vascular disorders [8, 9]. A previous study reported that PTX prevents non-alcoholic steatohepatitis (NASH)-related liver tumorigenesis through the attenuation of chronic hepatic inflammation in a mouse model [10]. In addition, PTX has been reported to suppress the synthesis of tumor necrosis factor (TNF)-α and oxidative stress and to improve the pathophysiological conditions in chronic inflammatory diseases [11, 12]. A recent paper reported that PTX has preventive effects on CRC development in a mouse model of azoxymethane (AOM)-induced and obesity-related colorectal carcinogenesis, primarily through the attenuation of inflammation and reduction of oxidative stress in the colonic mucosa [13].

Based on the studies mentioned above, we expected that PTX might have anti-inflammatory properties, which can contribute in attenuating chronic inflammation in the bowel and suppress CRC development in inflamed colon. To confirm this hypothesis, the potential chemopreventive ability of PTX was examined in a colitis-related mouse CRC model induced by AOM and dextran sodium sulfate (DSS) [14, 15]. In the present study, Kyoto adenomatous polyposis coli (APC) delta (KAD) rats, which have an *Apc* mutation and are susceptible to colorectal carcinogenesis [16], were employed and the development of colorectal tumorous lesions were evaluated. In addition, endoscopic examination of rat colon was performed to investigate its usability in observing and evaluating tumor progression and inflammation in the colorectum.

## RESULTS

### General observations

During the course of experiment, administration of PTX did not cause any clinical symptoms. The data for the weight of body, liver, kidneys, and spleen, and the length of the large bowels of the rats in all the groups at the end of the study are listed in Table 1. There were no significant differences in the weight of body and organs among the experimental groups. Moreover, there were no pathological alterations that would suggest toxicity of PTX in the liver, kidney, and spleen of rats (data not shown). The large bowel of rats in group 1, which were administered AOM plus DSS treatment, was significantly shorter compared to that in AOM/DSS-untreated group 5 (*P* < 0.05), but there is no significant difference among groups 1–3.

**Table 1:** Body, liver, kidneys, and spleen weights of the experimental rats

Group No.	Treatment		No. of rats	Body wt (g)	Length of colon (cm)	Relative wt (g/100 g body wt)		
	AOM/DSS	PTX (mg/l)				Liver	Kidneys	Spleen
1	+	−	12	310.7 ± 17.5^a^	17.5 ± 0.90^b^	3.7 ± 0.3	0.73 ± 0.04	0.22 ± 0.02
2	+	125	12	311.0 ± 15.4	18.1 ± 1.36	3.7 ± 0.2	0.74 ± 0.04	0.22 ± 0.01
3	+	500	12	302.0 ± 19.5	20.0 ± 4.03	3.7 ± 0.2	0.76 ± 0.06	0.24 ± 0.02
4	−	500	6	307.1 ± 6.1	23.0 ± 1.26	3.7 ± 0.1	0.72 ± 0.02	0.19 ± 0.01
5	−	−	6	300.2 ± 6.4	23.1 ± 0.49	3.6 ± 0.1	0.68 ± 0.04	0.20 ± 0.02

^a^Mean ± SD.

^b^Significantly different from Group 5 (*P* < 0.05).

### Endoscopic observation and evaluation of mucosal inflammation in the colorectum

**Figure 1:**
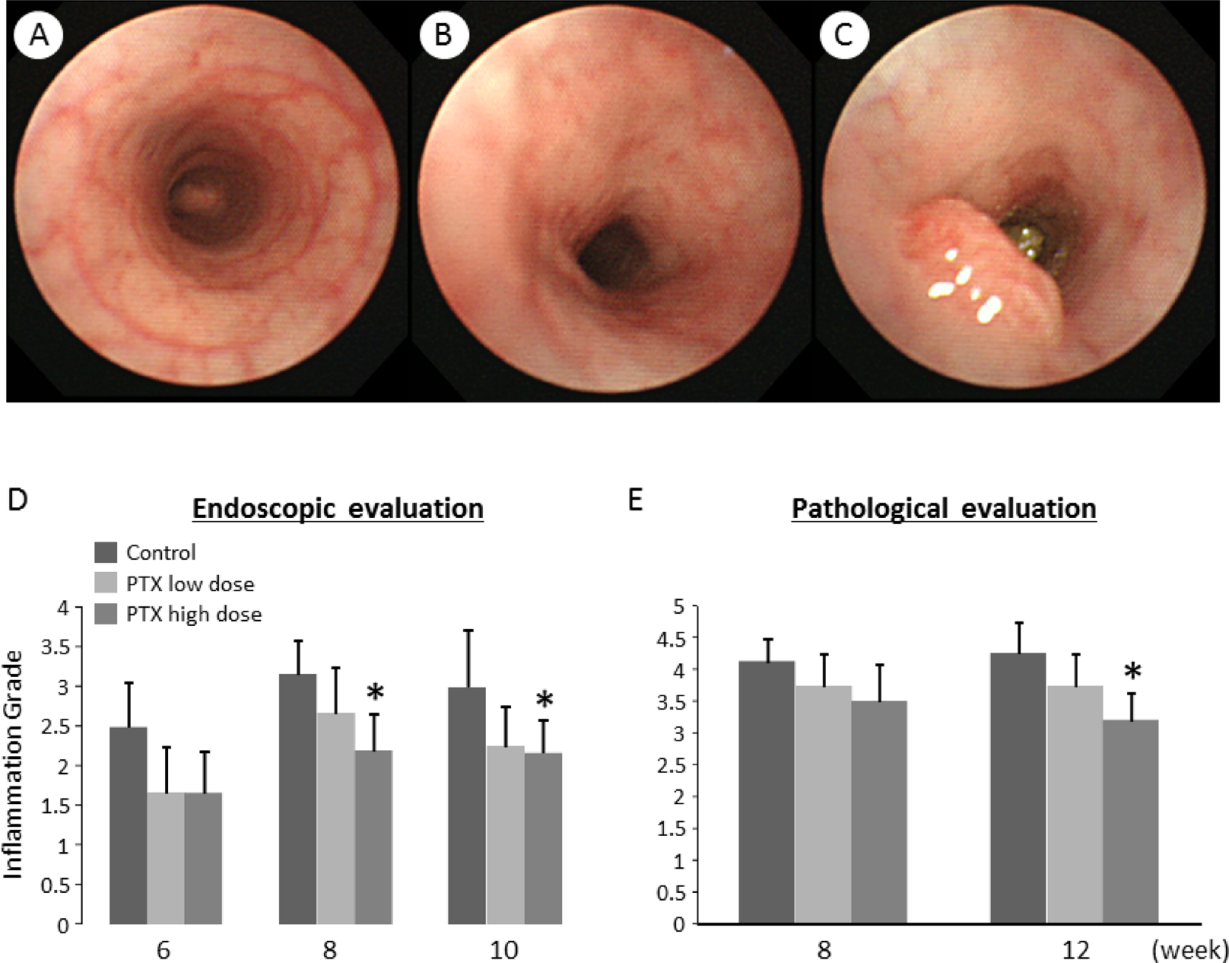
Endoscopic images and effects of pentoxifylline on AOM/DSS-induced inflammation and tumors in the colon of KAD rats (**A**–**C**) Endoscopic images of the colon. (A) Colonic mucosa considered as normal in non-treated group. (B) Inflamed colonic mucosa induced by AOM/DSS, characterized by mucosal reddening and edema and indistinct vascular pattern. (C) A colon tumor induced by AOM/DSS. Evaluation of colonic inflammation by (**D**) endoscopic and (**E**) pathological observation at indicated time points (weeks after AOM injection). The evaluation was based on the Matt's grade. Each column represents the mean ± SD. ^*^indicates statistically significant differences compared to AOM/DSS group; *P* < 0.05.

The endoscopic examination of the experimental rats was performed under inhalational anesthesia to evaluate mucosal inflammation and to observe tumor development in the colorectum. After proper preparation, we could observe colorectal mucosa and lesions, including polyps and tumors (Figure 1A–1C). The mucosal inflammation was evaluated endoscopically as per the Matts' Grade [17], which revealed that the inflammation grade was significantly lower in high-dose PTX-treated group than that in untreated group at weeks 8 and 10 (Figure 1D). In addition, colonic inflammation was also assessed pathologically with samples from endoscopic biopsy and autopsy, indicating that mucosal inflammation was attenuated in high-dose PTX-treated group at sacrifice compared to untreated group (Figure 1E).

### PTX-treated rats show decreased colon tumor development

**Figure 2:**
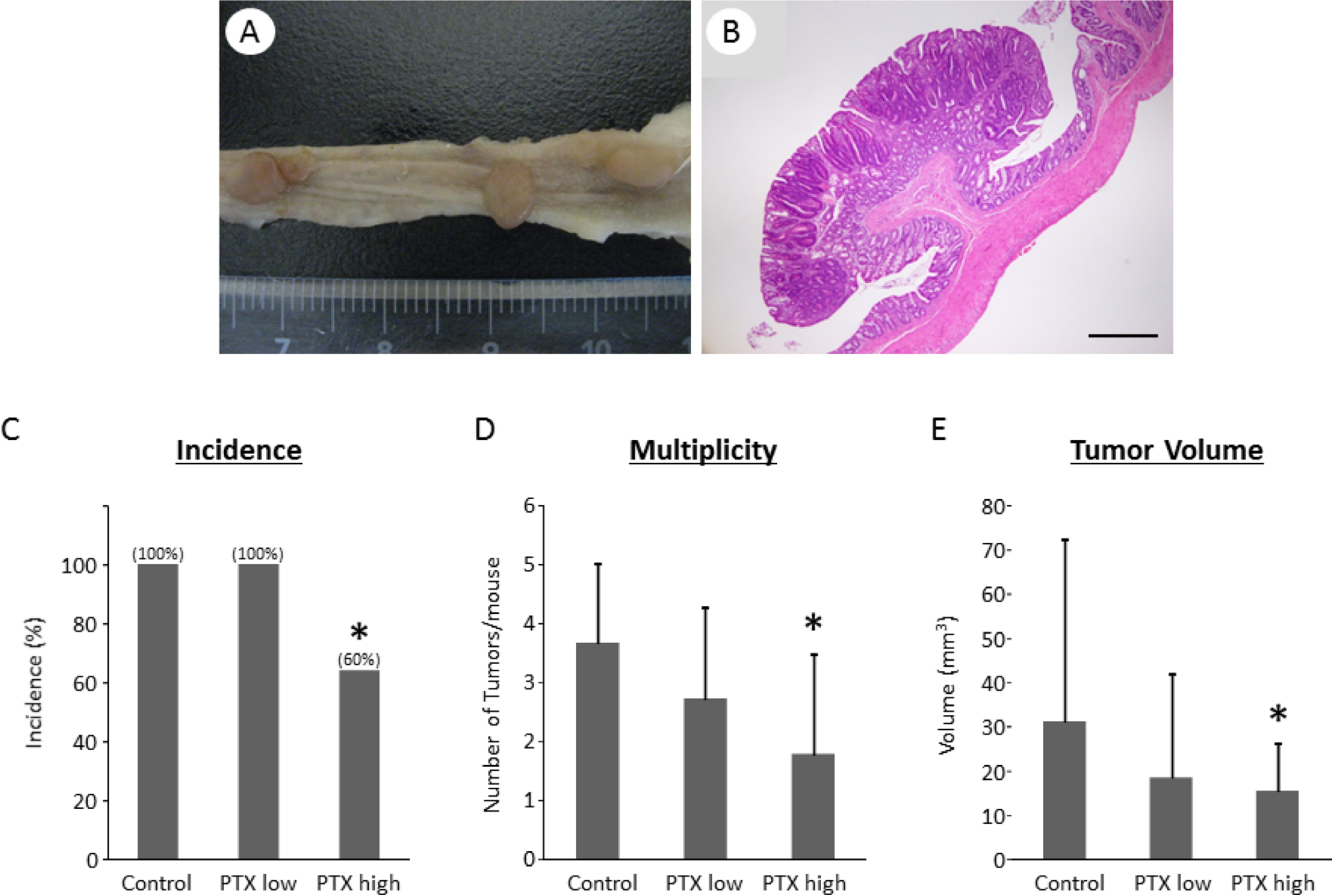
Effects of pentoxifylline on AOM/DSS-induced colon tumor in KAD rats (**A**) Macroscopic and (B) microscopic pictures of colon tumors developed in AOM/DSS-treated KAD rats. (**B**) Stained with hematoxylin and eosin. Scale bar, 500 μm. (**C**) Incidence, (**D**) multiplicity, and (**E**) tumor volume of colon tumors. (C–E) Each column represents the mean ± SD. ^*^indicates statistically significant differences compared to AOM/DSS group; *P* < 0.05.

Macroscopically, colon tumors were observed only in the colons of rats that received AOM and DSS (Figure 2A); therefore, rats in groups 1–3 were mainly analyzed hereafter. Histopathological examination revealed that the tumor nodules seen in the colons of the AOM/DSS-treated rats were considered as adenomas (Figure 2B). The histopathological changes induced by PTX treatment were not found. The rats treated with high dose of PTX were characterized by significantly lower incidence of colon tumor, fewer tumors, and smaller tumor volume compared to those in the AOM/DSS group (Figure 2C–2E). These findings suggest that PTX prevented AOM/DSS-induced colorectal carcinogenesis.

### Effects of PTX on oxidative stress in the experimental rats

**Figure 3:**
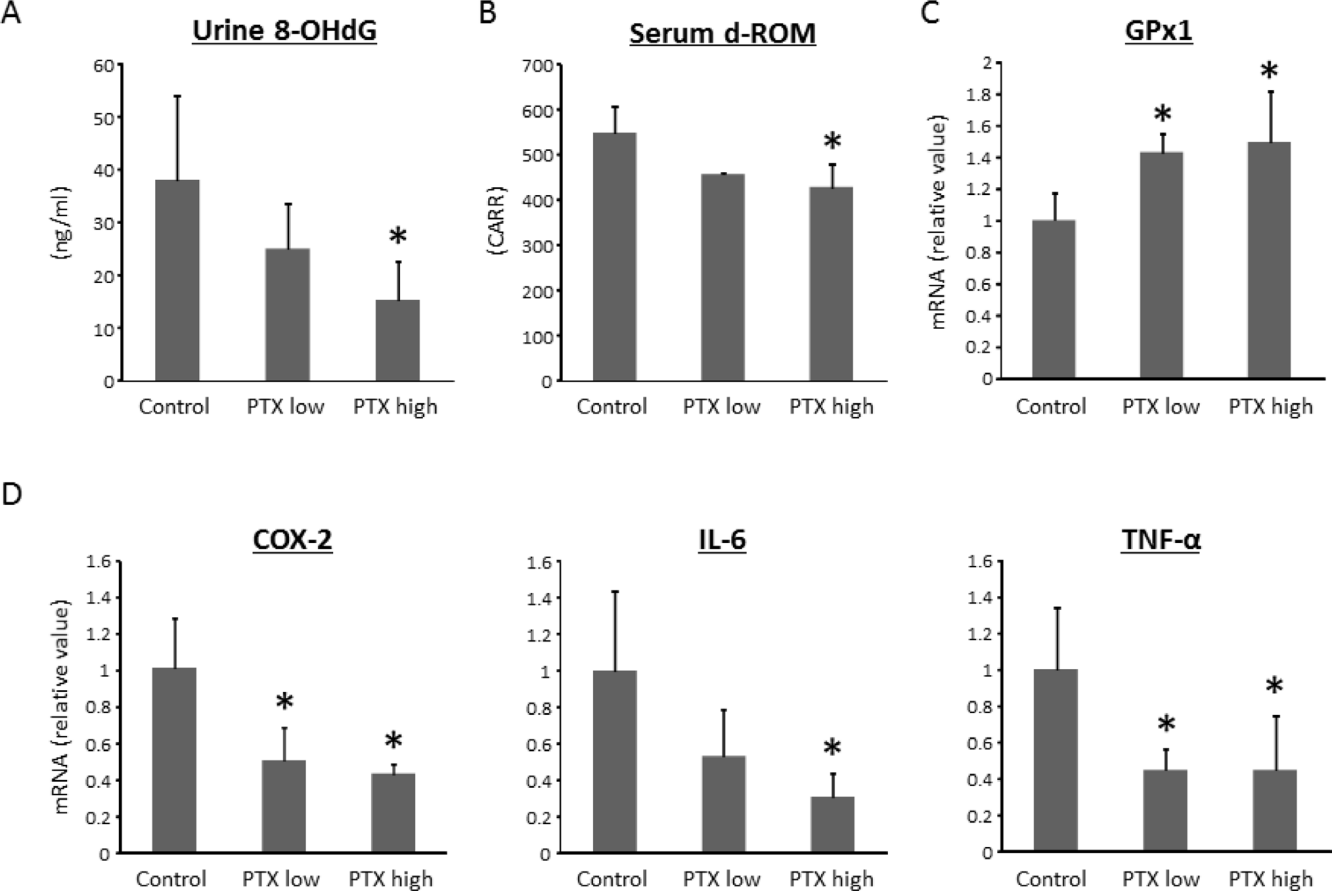
Effects of pentoxifylline on oxidative stress and colonic expression levels of pro-inflammatory cytokines in experimental rats (**A**) The levels of urinary 8-OHdG were measured by enzyme-linked immunosorbent assay. (**B**) Hydroperoxide levels in serum were determined by the d-ROM test. Total RNA was isolated from colonic mucosa of experimental rats, and the expression levels of (**C**) GPx1, (**D**) COX-2, IL-6, and TNF-α mRNA were examined by quantitative real-time RT-PCR using specific primers. Values are expressed as mean ± SD. ^*^indicates statistically significant differences compared to AOM/DSS group; *P* < 0.05. 8-OHdG, 8-hydroxydeoxyguanosine. d-ROM, derivatives of reactive oxygen metabolites.

Because oxidative stress is implicated in inflammatory bowel disease and colorectal tumorigenesis [18], we examined the severity of oxidative stress and the levels of an anti-oxidant biomarker in the experimental rats. To assess the systemic oxidative stress, we measured urinary 8-hydroxy-2′-deoxyguanosine (8-OHdG) levels, which reflect DNA damage induced by oxidative stress, and serum d-ROM, which is a marker for hydroperoxide content. The levels of 8-OHdG and derivatives of reactive oxygen metabolites (d-ROMs) were markedly decreased by high-dose PTX treatment (Figure 3A and 3B). The mRNA expression levels of glutathione peroxidase 1 (GPx1), which is an anti-oxidant enzyme, were significantly up-regulated in the colonic mucosa by administration of PTX (Figure 3C).

### Effects of PTX on the levels of pro-inflammatory cytokines in the colonic mucosa of experimental rats

Chronic inflammation plays a vital role in the pathogenesis of CRC [18, 19]. Therefore, the effects of PTX on the levels of pro-inflammatory cytokines, including cyclooxygenase (COX)-2, interleukin (IL)-6, and TNF-α, in the mucosa of colorectum were examined. The mRNA expression levels of COX-2, IL-6, and TNF-α in the colonic mucosa were significantly decreased by the PTX treatment, as shown in Figure 3D. These findings were consistent with evaluation of inflammation degree (Figure 3D and 3E).

### Effects of PTX on cell proliferation and NF-κB activity in the colonic mucosa

**Figure 4:**
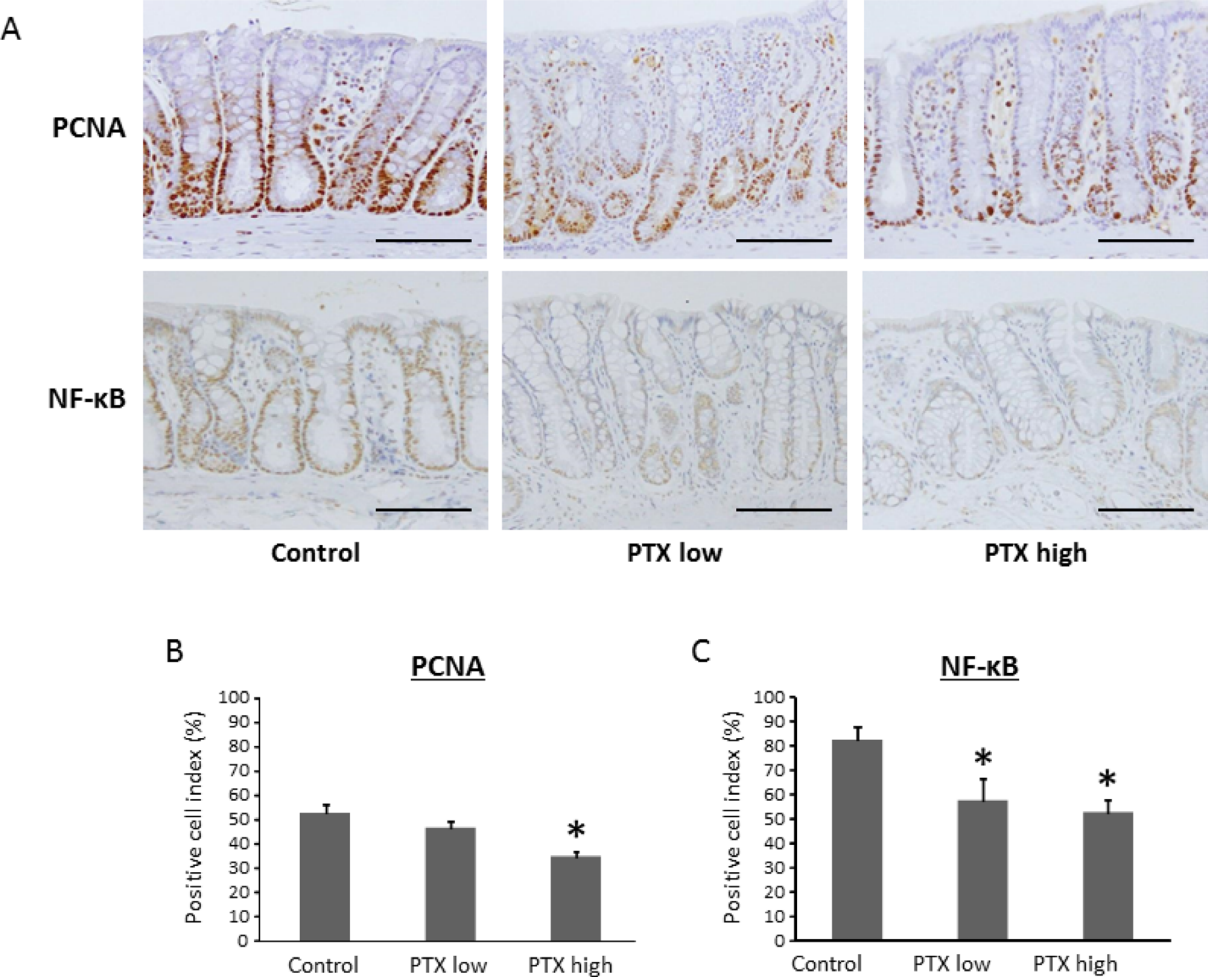
Effects of pentoxifylline on NF-κB activity and cell proliferative activity in colonic mucosa of experimental rats Sections of the colon were stained with anti-PCNA and anti-phosphorylated-NF-κB p65 antibodies. (**A**) Representative pictures from each group are shown. The positive cell indices were determined by counting (**B**) PCNA- and (**C**) NF-κB p65-positive cells in the colonic crypts. Scale bars, 100 μm. Each column represents the mean ± SD of triplicate assays. ^*^indicates statistically significant differences compared to AOM/DSS group; *P* < 0.05.

The activation of nuclear factor-κB (NF-κB) is significantly involved in the progression of inflammation and in the activation of cell proliferation in the colonic mucosa [20]. We, therefore, investigated the effects of PTX on cell proliferation and NF-κB activity in the colonic mucosa of the experimental rats. As shown in Figure 4A and 4B, PTX administration markedly decreased the proliferating cell nuclear antigen (PCNA)-labeling indices of non-lesional crypts. The indices of phosphorylated-NF-κB p65-positive cells were also significantly reduced by the PTX treatment (Figure 4A and 4C). PCNA and NF-κB were determined to be positive by nuclear staining. These observations indicate that PTX inhibited NF-κB activity and cell proliferation in the colonic mucosa of AOM/DSS-treated rats, which contributed to the reduction in the development of colon cancer.

## DISCUSSION

The process of CRC development is complicated, owing to the involvement of several factors, including genetic alterations, chronic inflammation, and carcinogen exposure [21]. The colorectal carcinogenesis model employed in this study, in which KAD rats were treated with AOM/DSS, appears to be highly useful in mimicking human colorectal carcinogenesis in inflamed colorectum, because this animal model involves *Apc* mutation, carcinogen exposure, and tissue inflammation [16, 22, 23]. In addition, this model is considered to be a useful tool for investigating the chemopreventive effects of several agents, because sequential endoscopic examination of colorectal tumor growth and inflammation of colonic mucosa can be performed [16, 22, 23]. In the present study, mucosal biopsies were also performed, which enabled us to do pathological evaluation of the degree of inflammation in colonic mucosa.

The prevention of CRC by administration of chemopreventive agents is a promising strategy, especially targeting chronic inflammation and oxidative stress, for those patients who have colorectal inflammation and are at increased risk for CRC [24, 25]. Among the candidates for CRC chemoprevention, COX-2 inhibitors, 5-ASA, and certain types of phytochemicals, such as green tea catechins, which possess anti-inflammatory and anti-oxidant effects, have shown promising results [26–28]. In this study, we focused on evaluating the effectiveness of PTX against CRC development, because it is reported to have anti-cancer activities besides being anti-inflammatory [10, 13, 29]. Moreover, because this compound has already been used as a drug, the feasibility of its clinical application is already established [8, 9]. The results of the present study clearly demonstrate that PTX effectively suppresses the colitis-related CRC development induced by the combined treatment of AOM and DSS in KAD rats. The PTX treatment decreased the incidence of colon cancer and the rate of occurrence of PCNA-positive intestinal epithelial cells compared to those in control rats, indicating that PTX suppressed colon tumorigenesis and intestinal epithelial cell proliferation. Although it may be interesting whether and how much PTX can suppress the development of colitis-related CRC in comparison with COX-2 inhibitors or 5-ASA, further studies are required using controlled experimental or clinical protocol in order to compare their effects.

KAD rats are known to display sustained inflammation in the colorectum and severe colitis as a result of DSS treatment [30]. In addition, this rodent is reported to show increased serum levels of COX-2 upon AOM/DSS administration [22]. These observations are important, because the increased level of COX-2 contributes to the development of CRC; therefore, targeting this molecule can be an effective strategy for chemoprevention of CRC [31, 32]. Using several colorectal carcinogenesis models, various agents, including COX-2 inhibitors, have been reported to inhibit the expression of COX-2, leading to the suppression of CRC development [13, 22, 28, 33]. In our study, PTX inhibited the expression of COX-2 in the colonic mucosa, which probably led to the suppression of colorectal carcinogenesis.

Chronic inflammation is often associated with enhanced oxidative stress, which leads to DNA damage and subsequent carcinogenesis [34]. Therefore, decreased levels of urine 8-OHdG and serum d-ROMs, which are markers for systemic oxidative stress, in PTX-treated groups, might contribute to the suppression of colitis-associated CRC development. These results are consistent with those of previous studies, demonstrating that loss of anti-oxidative stress potential enhances tumorigenesis [35, 36]. The mechanism by which PTX attenuates oxidative stress might be related to the upregulated levels of antioxidant enzymes, including GPx1, as was also observed in a previous study [37].

TNF-α is a major promoter of inflammation-related cancer development among different cytokines, which regulates the pathological condition of IBD [38, 39]. The transcription factor NF-κB is frequently found in inflamed colonic mucosa in patients with IBD, and activation of NF-κB is deeply associated with the induction of TNF-α [40]. Recent studies have demonstrated that PTX reduces the expression of TNF-α by inhibiting the NF-κB activity in various organs [41, 42]. In the present study, the expression of TNF-α and NF-κB was inhibited in the colorectum of the PTX-treated groups. PTX also suppresses the expression of IL-6, the other key cytokine in IBD pathogenesis [43].

This study has several limitations. It was impossible to obtain the data of incidence and multiplicity of rat tumors at weeks 6, 8 and 10 from endoscopic evaluation. This was because we could not observe endoscopically all area of colorectum in mice. In addition, the size of all tumors were not able to be measured endoscopically. If there were more suitable devices for observing colorectum of rodents, those parameters might be evaluated.

In conclusion, the results of this study support the possibility that the attenuation of chronic inflammation and oxidative stress by PTX could be a potentially effective strategy for chemoprevention of IBD-related CRC. Because PTX has been in clinical practice without severe side effects [44], further studies can be conducted relatively easily for investigating the usefulness of this agent in chemoprevention of CRC in patients with IBD.

## MATERIALS AND METHODS

### Animals, chemicals, and diets

Male KAD rats, aged 4 weeks (Charles River Japan, Tokyo, Japan), were maintained at the Gifu University Animal Facility according to the Institutional Animal Care Guidelines. All the rats were housed in plastic cages, with free access to drinking tap water and a pelleted basal diet (CRF-1; Oriental Yeast, Tokyo, Japan), under controlled conditions of humidity (50 ± 10%), light (12/12 h light/dark cycle) and temperature (23 ± 2°C). AOM, a colonic carcinogen, was purchased from Sigma Chemical Co. (St. Louis, MO, USA). DSS was purchased from MP Biomedicals, LLC (Aurora, OH, USA). For the induction of colitis, a 2.0% (w/v) DSS solution was prepared in distilled water. PTX was purchased from Sigma Chemical Co.

### Experimental procedure

Forty-eight male KAD rats were quarantined for 7 days, and then divided randomly into experimental and control groups. The rats in group 1 (*n* = 12) were given a single intraperitoneal injection of AOM (20 mg/kg body weight). Starting from 1 week after the injection of AOM, the rats received DSS (2.0% w/v) in drinking water for 7 days, and thereafter no further treatment was done until 17 weeks of age. The rats in groups 2 (*n* = 12) and 3 (*n* = 12) were treated with AOM and DSS; after 1 week of DSS treatment, the rats in these two groups were given water containing 125 and 500 mg/L of PTX, respectively. The rats in group 4 (*n* = 6) were given water containing 500 mg/L PTX concurrently with those in group 3. The rats in group 5 (*n* = 6) were not given any treatment and were used as control. The concentration of PTX was determined according to previous reports [45, 46]. All procedures, including the excision of large bowel, collection of blood samples from the inferior vena cava, and collection of urine from the bladder, were performed on rats that had been killed by CO_2_ asphyxiation at 17 weeks of age. The excised colons were carefully inspected and the visible tumors were analyzed. The tumor volume was calculated using the formula: (largest diameter) × (smallest diameter) × (smallest diameter) × 0.5. The colon was rolled like a “Swiss roll” [47] and paraffin-embedded sections were prepared using routine procedures for subsequent histopathological and immunohistochemical investigations. The histopathological grading of inflamed colonic mucosa was done as per the Matts' Grade [17].

### Endoscopic observation of colon and biopsy of mucosa

At the 6, 8, and 10 weeks of AOM injection (corresponding to 11, 13, and 15 weeks of age, respectively), endoscopic observations were made for sequential evaluation of inflamed mucosa and tumor development in the colorectum. The procedure involved, administration of inhalational anesthetic, isoflurane, after which the colon was flushed several times with enema of tap water until the feces in the range of observations were cleared. The fiberscope for human bronchi (BF-1T240, Olympus, Tokyo, Japan) was employed with the system (EVIS LUCERA CV-260, Olympus) and inserted into the colon and endoscopic images were obtained. Biopsies of the colonic mucosa were performed at the same time using appropriate biopsy forceps. The endoscopic grading of inflamed colonic mucosa was also done according to Matts' Grade [17].

### Immunohistochemical analyses

Immunohistochemical staining for PCNA and phosphorylated-NF-κB p65 were done on histological sections to estimate the cell proliferation and NF-κB activity, respectively, in the colorectal mucosa, using the labeled streptavidin-biotin method (LSAB kit; Dako, Glostrup, Denmark) with primary anti-PCNA antibody (1:100, Santa Cruz Biotechnology, Dallas, TX, USA) and anti- phosphorylated-NF-κB p65 antibody (1:50, Ser276; Cell Signaling Technology, Danvers, MA, USA). The positive cell indices (%) for PCNA and phosphorylated-NF-κB p65 were determined based on previous methods [48, 49].

### RNA extraction and quantitative real-time reverse transcription-PCR analysis

Total RNA was isolated from scraped colon mucosa of experimental rats using RNeasy Mini Kit (QIAGEN, Venlo, Netherlands). The cDNA was synthesized from 0.2 μg of total RNA using High Capacity cDNA Reverse Transcription Kit (Applied Biosystems, Foster City, CA, USA). A quantitative real-time reverse transcription-PCR (RT-PCR) analysis was performed using a LightCycler Nano (Roche Diagnostics, Indianapolis, IN, USA) with LightCycler 480 SYBR Green I Master Mix (Roche Diagnostics). The specific primers used for amplifying COX-2, GPx1, IL-6, TNF-α, and glyceraldehyde-3-phosphate dehydrogenase (GAPDH) genes were designed using the software available at the Roche Universal Probe Library Assay Design Center (https://lifescience.roche.com/global_en/brands/universal-probe-library.html). The sequences for these primers are shown in Table 2. The expression levels of *Cox-2*, *GPx1*, *IL-6*, and *TNF-*α were normalized to the expression level of *GAPDH*.

**Table 2:** Primer sequences

Target gene	5′-primer	3′-primer
Cox-2	TACACCAGGGCCCTTCCT	TCCAGAACTTCTTTTGAATCAGG
Gapdh	TGGGAAGCTGGTCATCAAC	GCATCACCCCATTTGATGTT
Gpx1	CGACATCGAACCCGATATAGA	ATGCCTTAGGGGTTGCTAGG
Il-6	CCCTTCAGGAACAGCTATGAA	ACAACATCAGTCCCAAGAAGG
Tnf-α	AGTTGGGGAGGGAGACCTT	CATCCACCCAAGGATGTTTAG

### Oxidative stress analysis

The 8-OHdG levels in urine were determined as a marker for oxidative stress using an enzyme-linked immunosorbent assay kit (NIKKEN SEIL, Shizuoka, Japan). The serum levels of hydroperoxide, which is also a marker for oxidative stress, were measured using the d-ROM test (FREE Carpe Diem, Diacron International s.r.l., Grosseto, Italy) [50].

### Statistical analysis

All the data are expressed as means ± SD. The differences between the groups were analyzed by two-way ANOVA. For significant differences indicated by ANOVA, the Tukey–Kramer multiple comparison test was performed. A value of *P* < 0.05 was considered to be significant.

## Abbreviations

AOM: azoxymethane
ASA: aminosalicylic acid
COX: cyclooxygenase
CRC: colorectal cancer
d-ROM: derivatives of reactive oxygen metabolite
DSS: dextran sodium sulfate
GAPDH: glyceraldehyde-3-phosphate dehydrogenase
GPx: glutathione peroxidase
IBD: inflammatory bowel diseases
IL: interleukin
KAD: Kyoto adenomatous polyposis coli delta
Kyoto APC: adenomatous polyposis coli
NASH: non-alcoholic steatohepatitis
NF-κB: nuclear factor-κB
8-OHdG: 8-hydroxy-2′-deoxyguanosine
PCNA: proliferating cell nuclear antigen
PTX: pentoxifylline
RT-PCR: reverse transcription-PCR
TNF: tumor necrosis factor
